# RNA from Trained *Aplysia* Can Induce an Epigenetic Engram for Long-Term Sensitization in Untrained *Aplysia*

**DOI:** 10.1523/ENEURO.0038-18.2018

**Published:** 2018-05-21

**Authors:** Alexis Bédécarrats, Shanping Chen, Kaycey Pearce, Diancai Cai, David L. Glanzman

**Affiliations:** 1 Department of Integrative Biology and Physiology, University of California, Los Angeles, Los Angeles, CA 90095; 2 Department of Neurobiology, David Geffen School of Medicine at University of California, Los Angeles, Los Angeles, CA 90095; 3 Integrative Center for Learning and Memory, Brain Research Institute, David Geffen School of Medicine at University of California, Los Angeles, Los Angeles, CA 90095

**Keywords:** *Aplysia*, epigenetics, learning and memory, memory transfer, RNA, sensitization

## Abstract

The precise nature of the engram, the physical substrate of memory, remains uncertain. Here, it is reported that RNA extracted from the central nervous system of *Aplysia* given long-term sensitization (LTS) training induced sensitization when injected into untrained animals; furthermore, the RNA-induced sensitization, like training-induced sensitization, required DNA methylation. In cellular experiments, treatment with RNA extracted from trained animals was found to increase excitability in sensory neurons, but not in motor neurons, dissociated from naïve animals. Thus, the behavioral, and a subset of the cellular, modifications characteristic of a form of nonassociative long-term memory (LTM) in *Aplysia* can be transferred by RNA. These results indicate that RNA is sufficient to generate an engram for LTS in *Aplysia* and are consistent with the hypothesis that RNA-induced epigenetic changes underlie memory storage in *Aplysia*.

## Visual Abstract

**Figure:**
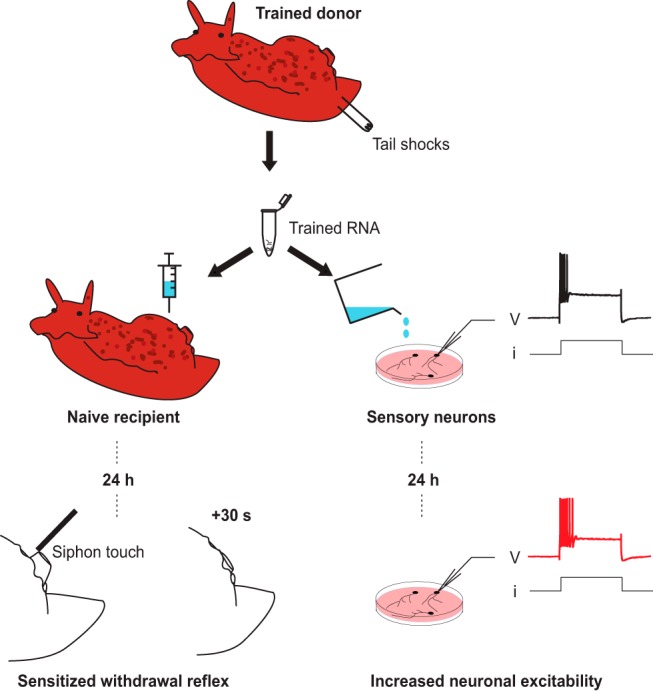

## Significance Statement

It is generally accepted that long-term memory (LTM) is encoded as alterations in synaptic strength. An alternative model, however, proposes that LTM is encoded by epigenetic changes. Noncoding RNAs (ncRNAs) can mediate epigenetic modifications. Therefore, RNA from a trained animal might be capable of producing learning-like behavioral change in an untrained animal. Here, it is demonstrated that the memory for long-term sensitization (LTS) in the marine mollusk *Aplysia* can be successfully transferred by injecting RNA from sensitized into naïve animals. Moreover, a specific cellular alteration that underlies sensitization in *Aplysia*, sensory neuron hyperexcitability, can be reproduced by exposing sensory neurons *in vitro* to RNA from trained animals. The results provide support for a nonsynaptic, epigenetic model of memory storage in *Aplysia*.

## Introduction

A major goal of modern neuroscience is to determine the identity of the engram, the physical memory trace (Semon, 1921). At present, it is widely accepted that long-term memory (LTM) is stored by learning-induced modifications of synaptic connections (Mayford et al., 2012; Takeuchi et al., 2014). But theoretical considerations (Holliday, 1999; Gallistel and Balsam, 2014) and recent experimental evidence (Chen et al., 2014; Johansson et al., 2014; Ryan et al., 2015) support the idea that LTM is stored within the cell bodies of neurons. Previously, it was reported that the memory for long-term sensitization (LTS) in *Aplysia* (Pinsker et al., 1973) involves an early, protein synthesis-dependent priming component that can persist independently of memory-related behavioral and synaptic alterations; the priming component permits LTM to be reinstated following its disruption by reconsolidation blockade, or to be induced by partial training after impairment of memory consolidation by retrograde amnesia (Chen et al., 2014; Pearce et al., 2017). The molecular identity of the memory priming component is unknown, but appears to involve epigenetic modifications (Zovkic et al., 2013). Noncoding RNAs (ncRNAs), which play important roles in memory formation (Rajasethupathy et al., 2009, 2012; Fiumara et al., 2015; Guven-Ozkan et al., 2016; Tan et al., 2017), represent a major mechanism for epigenetic alterations (Peschansky and Wahlestedt, 2014; Savell et al., 2016). This raises the intriguing possibility that constituents of LTM may be transferred from a trained to an untrained animal by RNA. Here, we tested this possibility in the case of LTS in *Aplysia*.

## Materials and Methods

### Behavioral training and testing

**Figure 1.**
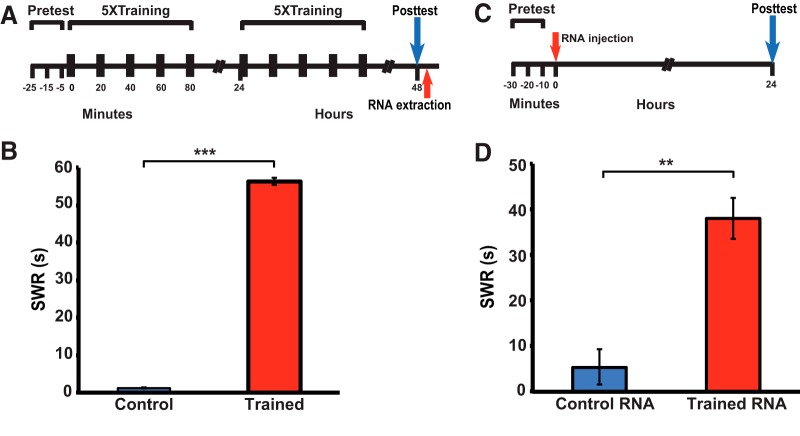
RNA extracted from sensitization-trained donor animals induces long-term enhancement of the SWR in recipient *Aplysia*. ***A***, Experimental protocol for inducing LTS in the donor animals. ***B***, Mean posttest duration of the SWR in the untrained control (1.2 ± 0.1 s, *n* = 31) and trained (56.4 ± 2.0 s, *n* = 34) groups. The trained group exhibited significant sensitization, as indicated by the comparison with control group (Mann–Whitney test, *U* = 496, *p* < 0.001). ***C***, Experimental protocol for the RNA injection experiments. The first pretest occurred 2–3 h after the posttest for the behavioral training (***A***). ***D***, Mean duration of the SWR measured at ∼24 h after the injection of RNA for the control RNA (5.4 ± 3.9 s, *n* = 7) and trained RNA (38.0 ± 4.6 s, *n* = 7) groups. The two groups differed significantly (*U* = 30, *p* < 0.003). Furthermore, Wilcoxon tests indicated that the difference between the pretest and posttest for the trained RNA group was significant (*W* = 28, *p* < 0.02), whereas it was not significant for the control RNA group (*p* > 0.2). The bar graphs in this and the following figures display means ± SEM; *p < 0.05, **p < 0.01, ***p < 0.001, n.s., nonsignificant.

**Figure 2.**
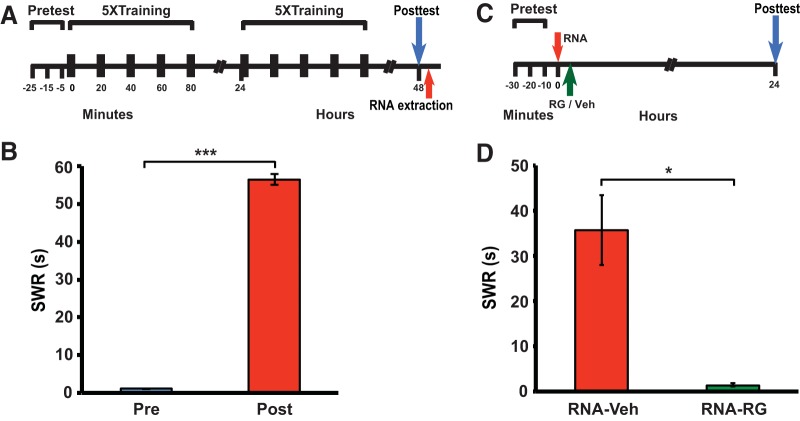
DNA methylation is required for RNA-induced enhancement of the SWR. ***A***, Experimental protocol for inducing sensitization in the second donor group. ***B***, Mean posttest duration of the SWR (*n* = 38). The training produced sensitization (mean posttest SWR = 56.4 ± 1.4 s, and mean pretest SWR = 1.1 ± 0.1 s; *W* = 741, *p* < 0.001). ***C***, Experimental protocol for testing the effect of DNMT inhibition on RNA-induced enhancement of the SWR. RG-108/vehicle was injected into animals 5–10 min after the RNA injection. ***D***, Mean postinjection duration of the SWR in the RNA-Veh (*n* = 3) and RNA-RG (*n* = 7) groups. The mean duration of the SWR in the RNA-Veh group (35.7 ± 7.7 s) was significantly longer than that in the RNA-RG group (1.4 ± 0.3 s; *U* = 27, *p* < 0.02). Moreover, the posttest SWR was sensitized compared to the pretest reflex in the RNA-Veh group (paired *t* test, *p* < 0.05), but not in the RNA-RG group (*p* > 0.4).

Adult *Aplysia californica* (80–120 g) were obtained from Alacrity Marine Biological Services and initially housed in a 50-gallon aquarium filled with cooled (12–14°C), aerated seawater. For the experiments, the animals were placed individually into custom-built Plexiglas chambers that were continuously perfused with cooled (14°C) seawater. One day before training, each animal was implanted bilaterally with Teflon-coated platinum wires (0.008-inch coated diameter, A-M Systems). For this procedure, the animal was anesthetized by cooling in cold seawater (4°C) for 13 min. Wires, prepared by removing the Teflon from the ends with forceps, were threaded through a 20-gauge needle, which was used to insert the wire into the animal’s tail. Following this procedure, the animal was placed into the experimental chamber, where it was given 24 h to recover and acclimate to the chamber. The siphon-withdrawal reflex (SWR) was tested as follows: The siphon was lightly stimulated with a soft, flexible probe and the duration of the resulting SWR was timed. Timing of the SWR began once the siphon had retracted completely beneath the parapodia and ended as soon as the siphon reappeared. Responses were given a score of 1.0 s if the siphon did not withdraw completely into the parapodia. Three pretests were delivered once every 10 min, beginning 25 min before the start of training (Figs. 1*A*, 2*A*). Sensitization training comprised two rounds of training separated by 24 h. Each round of training consisted of five bouts of tail shocks delivered at 20-min intervals. During each bout of training, the animal received three trains; the intertrain interval was 2 s. Each train was 1 s in duration and consisted of shocks (10-ms pulse duration, 40 Hz, 120 V) delivered to the animal’s tail via a Grass stimulator (S88, Astro-Med) connected to the platinum wires. A single posttest of the SWR, performed exactly as the pretests, was made at 48 h after the start of training. The testing and training were conducted by different experimenters, and the tester was blind to the experimental treatment of the animal.

In the experiments involving RNA injections (see Results), naïve animals were given three pretests, identical to those that preceded the sensitization training, at 30, 20, and 10 min before the injection (Figs. 1*C*, 2C). A single posttest of the SWR was performed at 24 h after the injection.

### RNA and drug preparation and injection

To prepare a single RNA injection, the pleural-pedal and abdominal ganglia were removed from four to five sensitization-trained animals, or from four to five untrained controls, immediately after the 48-h posttest. The total RNA was then extracted from the dissected ganglia. The ganglia were initially homogenized in TRIzol reagent for 30 s; typically, 1 ml TRIzol was used to homogenize the central ganglia from two animals. For every 1 ml TRIzol reagent, 200 μl chloroform was added and mixed by vortexing for 15 s. After incubation at room temperature for 5–10 min, the sample was centrifuged at 12,000 × *g* for 15 min. The upper aqueous phase was transferred into a new tube. The sample was then centrifuged for 10 min at 4°C after addition of 500 μl isopropanol to precipitate the RNA. The resulting RNA pellets were washed with 70% ethanol and centrifuged for 2 min at 4°C. After being air-dried for 10 min, the RNA pellet from each tube was dissolved in 30 μl DIH_2_O; then the RNA from ganglia dissected from trained animals (typically, from four animals) was combined, or the RNA from ganglia dissected from untrained animals was combined, into a single tube, and the RNA concentration was measured using Nano Drop (Thermo Fisher ND-1000). After the RNA concentration had been determined, 70 μg of the combined RNA was aliquoted and ASW was added to this aliquot to attain a volume of 100 μl; this solution was then injected into the hemocoel of an animal via its neck. Each recipient animal therefore received 70 µg of either RNA from trained animals or RNA from control animals.

The DNA methyltransferase (DNMT) inhibitor RG108 (Sigma) was dissolved in DMSO to a concentration of 25 mM. To inhibit DNMT, a volume of 100 μl/100 g of body weight of RG108 was injected intrahemocoelically into each animal (Fig. 2*C*).

### Cell culturing and electrophysiological measurements

Pleural sensory neurons and small siphon (LFS) motor neurons were individually dissociated from adult animals and placed into cell culture (Rayport and Schacher, 1986; Lin and Glanzman, 1994). Some of the cell cultures comprised isolated neurons, either exclusively sensory or exclusively motor neurons; others comprised synaptically coupled pairs of neurons, each consisting of a single sensory neuron and a single motor neuron. The cell culture medium was composed of 50% *Aplysia* sterile hemolymph and 50% Leibowitz-15 (L-15, Sigma). During electrophysiological recording the cell cultures were perfused with 50% ASW and 50% L-15 (recording medium). The recordings from isolated neurons were made using dissociated neurons that had been in culture for 5 d at the start of the experiments. For the experiments on synaptically coupled pairs of neurons (sensorimotor cocultures), the neurons were in culture for 3 d before the initial recordings. The neurons were impaled with sharp micropipettes (20–30 MΩ) filled with 1.5 M potassium acetate, 0.5 M potassium chloride, and 0.01 M HEPES (pH 7.2). The recorded voltage signals were amplified with an Axoclamp 2B amplifier (Molecular Devices), digitalized with an ITC-18 (Instrutech), and acquired and stored using Axograph software.

During the measurements of the biophysical properties of isolated sensory and motor neurons, the cell membrane potential was current clamped at –50 mV. The action potential (AP) firing threshold was determined by injecting 2-s current pulses of incremental intensity (0.1 nA for the sensory neurons and 0.01 nA for the motor neurons). Cells were injected with a 2-s steady pulse of suprathreshold positive current for the measurements of neuronal excitability (Liu et al., 2011). In the case of the sensory neurons, current pulses of 0.5, 1.0, or 2.0 nA were used depending on whether the initial firing threshold was <0.5, ≥0.5, or ≥1.0 nA, respectively. Sensory neurons were excluded from the analysis if their resting membrane potential was more depolarized than –35 mV. To test the excitability of motor neurons, positive current pulses of 0.1, 0.2, or 0.3 nA were used when the initial spike threshold was <0.1, ≥0.1, or ≥0.2 nA, respectively. Motor neurons whose membrane potentials were more depolarized than –30 mV were excluded. After the electrophysiological measurements were completed, the microelectrodes were removed from the neurons, and the cell cultures were treated with RNA-containing medium or vehicle solution (see Results). Twenty-four hours later, the neurons were reimpaled and their electrophysiological properties remeasured.

In the experiments involving sensorimotor cocultures, the amplitude of the monosynaptic EPSP evoked by a single presynaptic AP was assessed on day 1 of the experiment. For this purpose, the presynaptic sensory neuron and postsynaptic motor neuron in the coculture were impaled with sharp microelectrodes. To prevent the motor neuron from spontaneously firing during testing, the neuron’s membrane potential was held at –80 to –85 mV by passing negative current (0.3–0.8 nA) into the cell via the recording microelectrode using the bridge circuit of the amplifier. An initial EPSP was elicited through brief intracellular stimulation of the sensory neuron using a positive current pulse (20 ms, 0.2–0.8 nA). After the pretest, the microelectrodes were removed from the sensory and motor neurons, and the recording medium was replaced with cell culture medium. Then the coculture was treated either with RNA-containing medium or control medium (see Results). The sensory and motor neurons were reimpaled with microelectrodes and the amplitude of the monosynaptic EPSP reassessed 24 h later.

### RNA/vehicle treatment of cell cultures

Following the initial electrophysiological measurements on day 1, the recording medium was washed out with normal cell culture medium. The cultures were then randomly assigned to treatment with RNA from trained animals (trained RNA group), RNA from untrained animals (control RNA group), or vehicle. For the RNA treatments, 1 μg of RNA was added to each cell culture dish, yielding a concentration of 0.5 μg of RNA per 1 ml of cell culture medium. The RNA from the trained animals, the RNA from the control animals, or the vehicle was added to the cell culture dish and left in the dish for 24 h, after which it was washed out with the recording medium for 30 min, and the posttest electrophysiological measurements made.

### Statistical analyses

The statistical analyses of the data were performed using SigmaStat (Systat Software). Nonparametric tests were used to assess the statistical significance of differences whenever necessitated due to non-normality of the data or to the violation of the assumption of homogeneity of variance among experimental groups. Mann–Whitney *U* tests were used for comparisons of two independent groups. A paired *t* test or a Wilcoxon rank-sum test was used to compare two dependent groups. When three independent groups were involved, the significance of the overall group differences was initially assessed with a one-way ANOVA or a Kruskal–Wallis test. Given that the group differences were significant, Dunn’s *post hoc* tests were used for pairwise comparisons. Normality of the distribution were tested with a Shapiro–Wilk test. Levene's test centered to the mean (car package) was used with R software to test for homogeneity of variance in the synaptic experiments. All reported levels of significance represent two-tailed values. The statistical analyses are summarized in Table 1.

**Table 1.** Statistical table

	Data structure	Type of test	Power (α = 0.05)
a (Fig. 1B)	Non-normally distributed	Mann–Whitney test	Not applicable
b (Fig. 1D)	Non-normally distributed	Mann–Whitney test	Not applicable
c (Fig. 1D)	Non-normally distributed	Wilcoxon test	Not applicable
d (Fig. 1D)	Non-normally distributed	Wilcoxon test	Not applicable
e (Fig. 2B)	Non-normally distributed	Wilcoxon test	Not applicable
f (Fig. 2D)	Non-normally distributed	Mann–Whitney test	Not applicable
g (Fig. 2D)	Normally distributed	Paired *t* test	0.647
h (Fig. 2D)	Non-normally distributed	Wilcoxon test	Not applicable
i (Fig. 3B)	Non-normally distributed	Kruskal–Wallis test followed by Dunn’s test	Not applicable
j (Fig. 3D)	Non-normally distributed	Kruskal–Wallis test	Not applicable
k (Fig. 4B)	Non-normally distributed	Levene’s test	Not applicable
l (Fig. 4B)	Non-normally distributed	Kruskal–Wallis test	Not applicable

## Results

### Injection of RNA from sensitization-trained donor animals causes enhancement of the withdrawal reflex in untrained recipients

To generate the RNA used for memory transfer, individual *Aplysia* were given sensitization training consisting of spaced bouts of tail shocks for two consecutive days (Fig. 1*A*). The training produced clear LTS, as indicated by the significant enhancement of the SWR 24 h after the second day of training (48-h posttest) in the trained group of animals (Fig. 1*B*). Immediately after the 48-h posttest, RNA was extracted from the central nervous system (pleural, pedal and abdominal ganglia) of the control and trained animals. The extracted RNA was then injected intrahemocoelically into other naïve *Aplysia* (recipient animals; Fig. 1*C*). (Note that occasional batches of wild-caught *Aplysia* did not sensitize. The behavioral data from these animals were excluded from the analysis, and RNA was not extracted from them.) The duration of the SWR in the recipients was measured 24 h after the RNA injection. The SWR was significantly enhanced in the trained RNA group of animals compared to the control RNA group (Fig. 1*D*). Furthermore, a within-group comparison indicated that the posttest duration of the reflex was significantly longer than the pretest duration in the animals that received the injection of the RNA from trained donors; by contrast, the posttest SWR was not significantly prolonged compared to the pretest SWR in animals that received the injection of RNA from the untrained donors. Thus, only the RNA from sensitized animals appeared to induce reflex enhancement in the recipient snails.

### Inhibition of DNA methylation blocks the behavioral effect of RNA from sensitized donor animals in the recipients

Both the consolidation and maintenance of the LTM for sensitization in *Aplysia* depend on DNA methylation (Rajasethupathy et al., 2012; Pearce et al., 2017). To determine whether the RNA-mediated behavioral enhancement similarly required DNA methylation, we examined whether inhibiting DNA methylation disrupted the sensitizing effect of the RNA from trained animals. *Aplysia* were again given 2 d of sensitization training, which produced LTS, and afterward RNA was extracted from their central ganglia (Fig. 2*A*,*B*). The RNA was then injected into two groups of naïve snails; 5–10 min later, one of these groups (RNA-RG group) was also given an intrahemocoelic injection of the DNMT inhibitor RG-108 (Brueckner et al., 2005; Pearce et al., 2017), whereas the other (RNA-Veh group) was given an injection of the vehicle solution (Fig. 2*C*). The RNA-Veh group exhibited significant enhancement of the SWR 24 h later; by contrast, the RNA-RG group did not show behavioral enhancement (Fig. 2*D*). Therefore, DNA methylation is required for RNA-induced enhancement of the SWR, as it is for tail shock-induced LTS of the reflex (Pearce et al., 2017).

### RNA from sensitized animals induces increased excitability in sensory neurons dissociated from naïve animals

A significant advantage of *Aplysia* as a model system for mechanistic analyses of learning and memory is the wealth of extant knowledge regarding the biological bases of sensitization in this organism (Kandel, 2001; Byrne and Hawkins, 2015). Accordingly, we tested whether RNA extracted from sensitization-trained animals caused cellular alternations that mimic those known to result from repeated tail shocks. To ascertain whether the cellular changes induced by RNA from sensitized animals mimic shock-induced cellular changes, we made use of sensory and motor neurons of the withdrawal circuit in dissociated cell culture (Lin and Glanzman, 1994).

**Figure 3.**
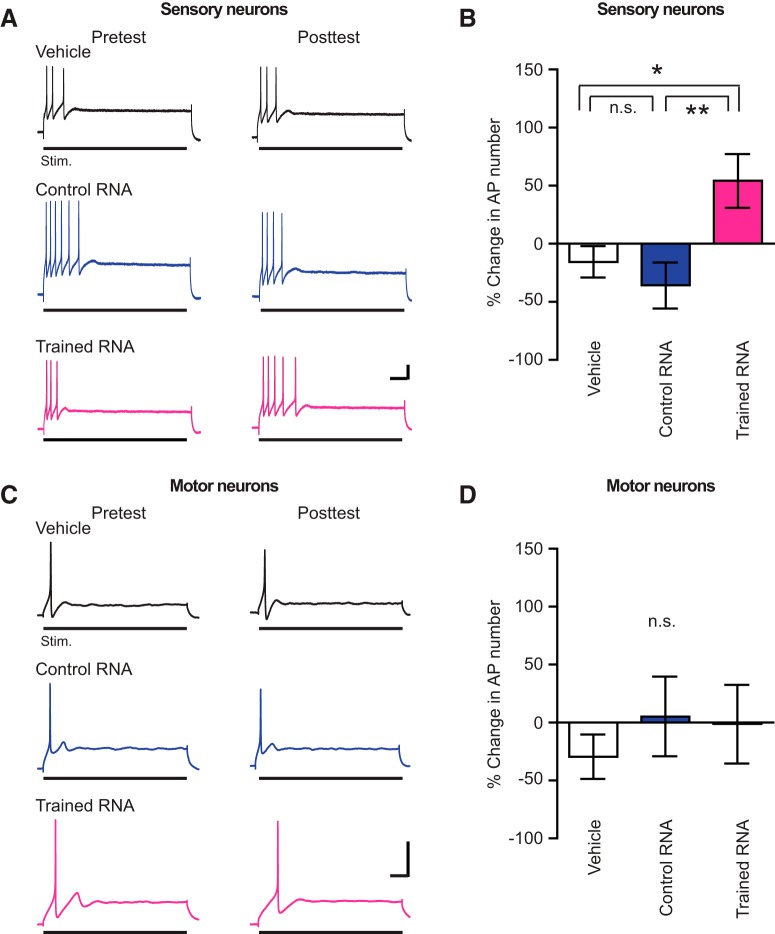
Treatment with RNA from trained animals increases excitability in dissociated sensory neurons but not in dissociated motor neurons. ***A***, Sample electrophysiological traces from excitability tests on sensory neurons. Scale bars: 20 mV, 0.25 s. ***B***, Changes in the excitability of the sensory neurons induced by RNA/vehicle treatment. The mean change in evoked APs in each group was: vehicle = –17.29 ± 12.86% (*n* = 19); control RNA = –35.76 ± 19.88% (*n* = 16); and trained RNA = 56.66 ± 22.07% (*n* = 19). The group differences were significant (Kruskal–Wallis; *H* = 11.81, *p* < 0.04). Dunn’s *post hoc* tests indicated that the increased firing in the trained RNA group was greater than that in the vehicle group (*q* = 2.44, *p* < 0.05) and control RNA group (*q* = 3.25, *p* < 0.004), respectively. The difference between vehicle and control RNA groups was not significant (*p* > 0.9). ***C***, Sample traces from tests of motor neuron excitability. Scale bars: 25 mV, 0.25 s. ***D***, Summary of posttreatment changes in the excitability of motor neurons. The mean changes were: vehicle group = –29.28 ± 19.16% (*n* = 15); control RNA group = 5.278 ± 34.36% (*n* = 12); and trained RNA group = –1.136 ± 34.01% (*n* = 14). The group differences in excitability were insignificant (*p* > 0.7).

In response to a prolonged pulse of depolarizing intracellular current, *Aplysia* sensory neurons exhibit spike “accommodation”: they fire at the beginning of, but not throughout, the current pulse (Klein et al., 1986). Long-lasting sensitization of the defensive withdrawal reflex is accompanied by a long-term increase in the excitability of the somata of central sensory neurons in the withdrawal circuit (Walters, 1987); this enhanced excitability is reflected as anti-accommodation, an increase in the number of APs evoked by a prolonged pulse of positive current (Cleary et al., 1998). To test whether RNA extracted from trained *Aplysia* alters sensory neuron accommodation, we used isolated sensory neurons in dissociated cell culture. The neurons were initially impaled with sharp microelectrodes and the number of APs evoked by a 2-s intracellular pulse of suprathreshold positive current quantified (Fig. 3*A*). Following this pretest, the sensory neurons were treated for 24 h with RNA from trained donors or RNA from untrained donors. Other sensory neurons were treated with an equivalent amount of the vehicle alone. The next day, the RNA/vehicle was washed out of the culture dishes with cell recording medium, and the neurons were reimpaled and reinjected with the same suprathreshold current to measure potential changes in excitability. The current injections produced significantly more APs in sensory neurons treated with RNA from sensitized animals than in sensory neurons treated with either vehicle or RNA from control animals (Fig. 3*B*). There was no significant difference in excitability between the sensory neurons treated with control RNA and those treated with the vehicle. Anti-accommodation is known to result from a decrease in cyclic AMP-dependent potassium currents in *Aplysia* sensory neurons, and, in particular, to reduction of the slowly-inactivating S-type current (Klein et al., 1986; Goldsmith and Abrams, 1992); thus, the RNA from sensitization-trained animals may enhance the excitability of sensory neurons through modulation of the same current that is modulated by electrical shocks to the body wall of *Aplysia*.

### RNA from sensitized animals does not increase the excitability of dissociated motor neurons

To ascertain the specificity of the cellular effects of the RNA treatment, we examined the effects of applying RNA from trained or control animals to isolated small siphon (LFS) motor neurons in dissociated cell culture. A previous study of LTS in *Aplysia* showed that, in contrast to the effects observed in sensory neurons, in motor neurons LTS was not accompanied by a significant increase in the number of APs evoked to intracellular injection of a prolonged pulse of suprathreshold current (Cleary et al., 1998). Thus, the induction of LTS does not produce an overall increase in the excitability of motor neurons. Similarly, we observed no effect of the RNA from sensitization-trained animals on excitability-related properties of isolated motor neurons in cell culture (Fig. 3*C*,*D*). This result indicates that the modulation of neuronal excitability by RNA from sensitized animals was specific to the sensory neurons.

### RNA from sensitized animals has a variable effect on synaptic strength in sensorimotor cocultures

**Figure 4.**
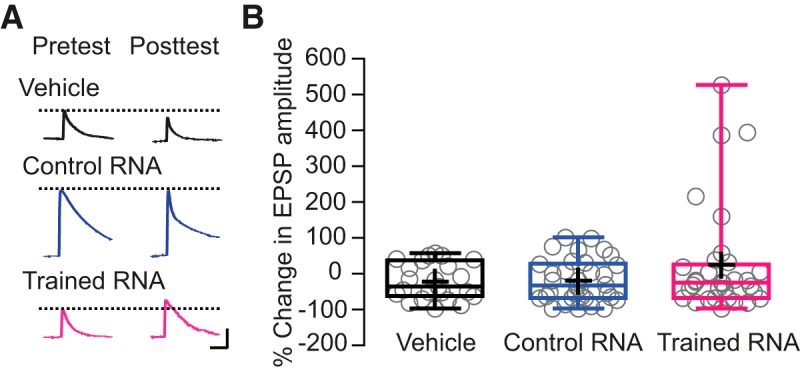
Exposure of *in vitro* sensorimotor synaptic connections to RNA from trained animals enhanced the strength of a subpopulation of synapses. ***A***, Representative records of EPSPs evoked in motor neurons by a single presynaptic AP before and 24 h after the RNA/vehicle treatments. Scale bars: 5 mV, 0.1s. ***B***, Box and whiskers plots showing the distribution of posttreatment changes in EPSP amplitude in the three experimental groups. The boxes delineate the second and third quartiles, the horizontal lines in the boxes represent the medians, and the vertical bars (whiskers) show the extent of the data spread. The crosses indicate the means, whereas individual data points are represented by circles. Mean posttreatment changes in EPSP amplitudes were: vehicle group = –23.38 ± 10.59% (*n* = 23); control RNA group = –21.32 ± 10.23% (*n* = 34); and trained RNA group = 22.71 ± 26.70% (*n* = 32). A Kruskal–Wallis test revealed no significant differences among the groups with respect to the mean changes in EPSP amplitude (*p* > 0.8). Note, however, that five of the 32 synapses treated with RNA from trained animals showed an increase of >150%, whereas none of the synapses treated with vehicle or RNA from control animals showed an increase of this magnitude. A Levene’s test confirmed that the three groups displayed significantly unequal variances (*F*_(2,86)_ = 5.883, *p* < 0.005).

LTS in *Aplysia* involves long-term facilitation (LTF) of the monosynaptic connection between the sensory and motor neurons of the withdrawal circuit (Frost et al., 1985). Accordingly, we examined the effects of RNA from trained and untrained donors on the strength of sensorimotor synapses in dissociated cell culture (Montarolo et al., 1986; Cai et al., 2008). There was no long-term effect of 24-h incubation with RNA from trained animals, RNA from control animals, or the vehicle on the mean EPSP evoked in the postsynaptic motor neurons by a presynaptic AP (Fig. 4). Nonetheless, although the mean EPSPs in the three experimental groups did not differ significantly, the variances among the EPSPs in the three groups were significantly unequal due to the greater variance in the EPSPs for the synapses treated with RNA from sensitization-trained animals. Inspection of the synaptic data revealed that the RNA from trained donors produced large enhancement of a subset of the sensorimotor synapses. Such enhancement was never observed for synapses treated with RNA from untrained animals or for synapses treated with the vehicle.

## Discussion

We have shown that RNA from sensitization-trained *Aplysia* contains critical components of the engram for LTS, as indicated by its ability to induce sensitization-like behavioral enhancement when injected into naïve recipient animals. Importantly, the RNA-induced sensitization, like the LTS induced by noxious stimulation, requires DNA methylation for its consolidation (Pearce et al., 2017; Fig. 2). Several of our cellular and behavioral results further argue that this putative transference of memory from donor animals to the recipients cannot be easily ascribed to nonspecific effects of the donor RNA. First, the control RNA (RNA extracted from untrained donors) did not produce sensitization of the SWR (Fig. 1). Second, the RNA from trained donors had an opposing effect on the excitability of cultured sensory neurons from that of untrained donors (Fig. 3*A*,*B*). Third, the changes produced by the RNA from sensitized *Aplysia* were selective for sensory neurons; the biophysical properties of motor neurons were unaltered by the RNA from sensitized donors (Fig. 3*C*,*D*). Admittedly, the alterations we observed in the biophysical properties of cultured sensory neurons after treatment with RNA from sensitized animals are unlikely to fully account for the behavioral changes produced in the intact recipient animals by injections of RNA from trained donors; nonetheless, because these biophysical alterations mimic those found in intact animals after LTS training (Walters, 1987; Cleary et al., 1998), they would be expected to contribute substantially to the RNA-induced sensitization.

It is interesting that the RNA from sensitization-trained animals appeared to produce strong facilitation only in a subset of sensorimotor synapses (Fig. 4). We do not understand the reason for the variability of the synaptic effect of the RNA from trained animals. One possibility is that there is an as-yet unappreciated inhomogeneity among the population of pleural sensory neurons and/or small siphon motor neurons that were used for the sensorimotor cocultures; according to this idea, only some of the dissociated neurons had the capacity to express the long-term changes that contribute to LTF. Another possibility is that the epigenetic alterations, particularly DNA methylation, that result from treatment with the RNA from sensitized animals more reliably induce cell-wide alterations, such as changes in intrinsic neuronal excitability (Meadows et al., 2016; see also Meadows et al., 2015), than synapse-specific LTF. Of course, these possibilities are not mutually exclusive.

Overall, the cellular changes caused by the RNA from trained animals were admittedly modest compared to the behavioral changes. But this is not unexpected; the defensive withdrawal reflexes in *Aplysia* are regulated by interneuronal neural circuits, in addition to the monosynaptic sensorimotor connections (Cleary et al., 1995). Injections of the RNA from sensitized donors may well have produced modifications of interneuronal pathways within the animals that contributed to behavioral sensitization. In addition, it is important to note that the RNA was removed from the donors 48 h after training; indeed, the RNA from trained animals produced a greater increase in the excitability of cultured sensory neurons at 48 h posttraining than long-term training with serotonin (Liu et al., 2011, their Fig. 6).

Our data indicate that essential components of the engram for LTM in *Aplysia* can be transferred to untrained animals, or to neurons in culture, via RNA. This finding raises two questions: (1) Which specific RNA(s) mediate(s) the memory transfer? and (2) How does the naked RNA get from the hemolymph/cell culture medium into *Aplysia* neurons? Regarding the first question, although we do not know the identity of the memory-bearing molecules at present, we believe it is likely that they are ncRNAs. Note that previous results have implicated ncRNAs, notably microRNAs (miRNAs) and Piwi-interacting RNAs (piRNAs; Rajasethupathy et al., 2009, 2012; Fiumara et al., 2015), in LTM in *Aplysia*. Long ncRNAs (lncRNAs) represent other potential candidate memory transfer molecules (Mercer et al., 2008). Regarding the second question, recent evidence has revealed potential pathways for the passage of cell-free, extracellular RNA from body fluids into neurons. Thus, miRNAs, for example, have been detected in many different types of body fluids, including blood plasma; and cell-free extracellular miRNAs can become encapsulated within exosomes or attached to proteins of the Argonaut (AGO) family, thereby rendering the miRNAs resistant to degradation by extracellular nucleases (Turchinovich et al., 2012, 2013). Moreover, miRNA-containing exosomes have been reported to pass freely through the blood-brain barrier (Ridder et al., 2014; Xu et al., 2017). And it is now appreciated that RNAs can be exchanged between cells of the body, including between neurons, via extracellular vesicles (Smalheiser, 2007; Valadi et al., 2007; Tkach and Théry, 2016; Ashley et al., 2018; Pastuzyn et al., 2018). If, as we believe, ncRNAs in the RNA extracted from sensitized animals were transferred to *Aplysia* neurons, perhaps via extracellular vesicles, they likely caused one or more epigenetic effects that contributed to the induction and maintenance of LTM (Fig. 2).

There have been prior reports of the successful transfer of LTM from trained donor animals to naïve recipients via cannibalism (McConnell, 1962) or RNA injection (Babich et al., 1965; Jacobson et al., 1965; Albert, 1966; Braud, 1970). However, these early claims have long been viewed with skepticism due to numerous failures to replicate the memory transfer effect (Hartry et al., 1964; Gross and Carey, 1965; Byrne et al., 1966; Luttges et al., 1966; Walker, 1966; Walker and Milton, 1966; McGaugh, 1967). The negative results convinced many that the positive reports of memory transfer were attributable to lack of proper controls for training-induced factors such as stress or arousal, and/or the influence of poorly defined aspects of the experimental methods used (time between the RNA injection and behavioral testing of the recipients, specific method of RNA extraction, etc.; McGaugh, 1967; Setlow, 1997).

A major advantage of our study over earlier studies of memory transfer is that we used a type of learning, sensitization of the defensive withdrawal reflex in *Aplysia*, the cellular and molecular basis of which is exceptionally well characterized (Kandel, 2001; Kandel, 2012; Byrne and Hawkins, 2015). The extensive knowledge base regarding sensitization in *Aplysia* enabled us to show that the RNA from sensitized donors not only produced sensitization-like behavioral change in the naïve recipients, but also caused specific electrophysiological alterations of cultured neurons that mimic those observed in sensitized animals. The cellular changes observed after exposure of cultured neurons to RNA from trained animals significantly strengthens the case for positive memory transfer in our study.

Another difference between our study and earlier attempts at memory transfer via RNA is that there is now at hand a mechanism, unknown 40 years ago, whereby RNA can powerfully influence the function of neurons: epigenetic modifications (Qureshi and Mehler, 2012). In fact, the role of ncRNA-mediated epigenetic changes in neural function, particularly in learning and memory, is currently the subject of vigorous investigation (Landry et al., 2013; Sweatt, 2013; Fischer, 2014; Nestler, 2014; Smalheiser, 2014; Marshall and Bredy, 2016). Our demonstration that inhibition of DNA methylation blocks the memory transfer effect (Fig. 2) supports the hypothesis that the behavioral and cellular effects of RNA from sensitized *Aplysia* in our study are mediated, in part, by DNA methylation (Rajasethupathy et al., 2012; see also Pearce et al., 2017).

The discovery that RNA from trained animals can transfer the engram for LTS in *Aplysia* offers dramatic support for the idea that memory can be stored nonsynaptically (Holliday, 1999; Gallistel and Balsam, 2014; Queenan et al., 2017), and indicates the limitations of the synaptic plasticity model of LTM storage (Mayford et al., 2012; Takeuchi et al., 2014). In addition, our results suggest that RNA could eventually be used to modify, either enhance or depress, memories.

## Synthesis

Reviewing Editor: Elizabeth Cropper, Mt Sinai Sch Med Decisions are customarily a result of the Reviewing Editor and the peer reviewers coming together and discussing their recommendations until a consensus is reached. When revisions are invited, a fact-based synthesis statement explaining their decision and outlining what is needed to prepare a revision will be listed below. The following reviewer(s) agreed to reveal their identity: Klaudiusz Weiss, Thomas Abrams

Both reviewers felt that this is an exciting study that could have a major impact on the field. Neither reviewer felt that additional experimentation is required. However, both reviewers would like to see sections of the manuscript re-worked. For example, both reviewers felt that the Discussion should be streamlined (as is described in more detail below). More specifically both reviewers felt that the ‘historical’ section of the Discussion should be greatly shortened. Further, they felt that sections of the manuscript are misleading in that they seem to imply that memory is transferred between individuals under physiological conditions. These sections of the manuscript should be rewritten.

Specific comments are below:

Reviewer #1

This is a powerful study of an inter-animal transfer of long term memory between animals. The Authors used sensitization training in Aplysia. They demonstrated that the enhancement of the siphon reflex produced by sensitization can be transferred to naïve animals by injecting them with RNA extracted from sensitized animals. Consistent, with this finding they show that ‘trained’ RNA increased the excitability of sensory neurons that drive the motor-neurons in the reflex they study. They used the DNA methyltrasferaze inhibitor, RG-108, to provide evidence for an involvement of epigenetic mechanisms in the memory transfer.

Overall, the quality of experimental designs, control procedures and statistical analyses is excellent.

I have only minor questions.

Pleural sensory neurons are nor a homogenous population. Have you made an attempt to select a specific subpopulation of sensory neurons for your electro-physiological experiments?

Was the RNA you injected into an individual animal derived from one or multiple donors?

In the Methods, to help your readers to follow the training procedures, refer them to diagrams in Figs. 1 & 2.

The Discussion is verbose. This is particularly obvious since a substantial part of the discussion deals with issues that were not investigated in this paper, e.g., transport of RNA from extracellular space into the neurons.

Reviewer #2

This manuscript presents very exciting results suggesting that RNA from the CNS of long-term sensitized Aplysia can initiate long-term sensitization in naive animals. Because RNA could migrate between neurons, e.g. via exosomes, this study could have an important impact on the field. However, several aspects of the presentation are substantially suboptimal; these could be easily improved to strengthen the ms.

Major comments.

1. Key take home message from the study: “the engram for sensitization in Aplysia ... can be transferred between conspecifics via RNA.” Note, this phrasing appears a second time - “the engram for LTM in Aplysia can be transferred between conspecifics via RNA.” This is much like Otto Loewi concluding from his classic experiment that modulation of the heart can be shared among frogs (or possibly among people). Even the use of the term ‘conspecifics’ actually confuses the message. The key results of this study are not about information sharing among conspecifics - analogous to pheromones. Rather the statement cited here simply refers to an experimental manipulation, so the term ‘conspecific’ is totally inappropriate. We would not describe cocultures where an L7 motor neuron and a sensory neuron come from different age animals as forming a neural circuit across conspecific animals. The last sentence in the Abstract exhibits the same problem: “We propose that the RNA from trained animals induces epigenetic changes in the nervous system of naïve animals, and that these changes constitute an essential part of the engram for long-term sensitization”. The transfer from trained to naive animals is not a mechanism that the authors propose, but rather a statement of the key experimental observation. Similarly in the Significance Statement “Our results indicate that RNA can mediate inter-individual transmission of the engram” again points to pheromone like signaling. Even the title ‘Transfer of the Engram’ suggests what is of interest is transfer of memory among individuals. I had to make a note at the outset that we are talking here about interneuronal signaling not about inter-individual communication or epigenetic transmission of ‘culture.’ The authors should decide whether they are more interested in impacting our understanding of biology or creating a catchy title that may, upon serious consideration, lead to disappointment.

2. Discussion of synaptic results. The highly variable effect of RNA from trained animals on the sensory neuron to motor neuron in culture is puzzling and merits more effective presentation and discussion. Five synapses out of 32 exhibited large increases of more than 2-fold to 5-fold in amplitude. The other synapses showed a modest increase or no increase. In contrast, the two groups of control synapses showed on average a decrease, and none of these connections increased more than two fold. How should this variability be interpreted? It is in marked contrast to the more than 7-fold increase in siphon withdrawal duration with injection of RNA from trained animals in Figs. 1 and 2. One possibility is that the behavioral changes in the siphon withdrawal response are mediated by neuronal changes at loci beyond the sensory neuron to motor neuron synapse. It has long been appreciated, though rarely discussed, that a prolonged withdrawal response of tens of seconds can not be directly mediated by an increase in the amplitude of the sensory neuron to motor neuron connection when EPSPs last at most a several hundreds milliseconds. Another possibility is that cell-wide, nucleus-dependent changes that are initiated by RNA from trained animals require local signals at synaptic sites for stabilization, much as Casadio et al (1999) described. (This possibility would not explain why behavioral sensitization with RNA injection is so robust.)

3. The difference in the effect of the RNA from trained animals on excitability in sensory neurons and on the sensory neuron to motor neuron EPSP merits more discussion. The increase in excitability represents the single most sensitive electrophysiological change associated with sensitization. The Walters 1987 paper, which is cited, shows an increase from 1 to >20 action potentials. A 6-fold increase in spike number with 5HT exposure is very common. In contrast, long-term facilitation at this synapse frequently exhibits a 60-100% increase. The 50% increase in action potential number in Fig. 3B, although significant, is a very modest increase. Thus, the synapses that do show increased strength have a relatively much stronger response than the sensory neurons exhibiting an increase in excitability. Put differently, the differences in the effects on excitability and synaptic strength are less puzzling than the authors acknowledge.

4. A great deal of the discussion is spent on historic results that failed to replicate. This begins with “There have been prior reports of the successful transfer of LTM from trained donors ...” By the second paragraph we are told “With hindsight, however, the failures to observe the memory transfer effect are perhaps not surprising.” “Lack of

modern scientific knowledge hindered the acceptance of the idea of memory transfer ...” The implication here is that a real phenomenon was somehow missed. This discussion begins to confound a lack of an effect with failure to understand mechanisms for such an effect, had it existed - “miRNAs, for example, have been detected in many different types of body fluids, including blood plasma; and cell-free extracellular miRNAs can become encapsulated within exosomes or attached to proteins of the Argonaut (AGO) family...” This would be relevant had there been a consistent effect of RNA in these earlier studies. This section, which spans more than 3 pages, is pretty much irrelevant. Many published findings don't replicate, and it is rather pointless to speculate about this - unless later experiments in the same system replicate the initial findings. Possible mechanisms that would be relevant for interpreting the present results are somehow discussed interspersed with this commentary on the earlier results, diluting the impact of this discussion. The failure of early results to replicate would be more appropriately covered in 2-3 sentences. The fact that naked RNA, which may have been dissociated from RNA-protein complexes (such as with Argonaute) or exosomes in Trizol, is still active in Aplysia is worth discussing, separate from commentary on early negative results. With all of this discussion of early non-findings, important points, such as the role of DNA methylation implicated in the present experiments, are not adequately emphasized.

Minor points.

5. Abstract “In additional cellular experiments” ‘Additional’ is ambiguous here, as these are the only cellular experiments so far.

Methods.

6. We are told the three specific periods in which animals were obtained, spanning 6 months over 2 years. This is of no benefit in reproducing the results, as we can't go back in time. Unless there is a seasonal effect, this information is not helpful.

7. “Trains of shocks spaced 2s apart.” Is this a 2 second ITI or is there a 2 second gap between trains?

8. Preparation of RNA. There is no information on the volumes of Trizol used, particularly for each CNS.

9. “70 μg of RNA was dissolved in dH2O” What was the volume of water? This spelling dH2O, as opposed to DIH2O, appears in a few places. ‘was’ should be ‘were.’

10. Current for testing excitability. This is a complicated protocol. ‘Respectively’ used in the motor neuron description would also be helpful for the sensory neuron description. In the case of motor neurons, it would be helpful to indicate these currents were for testing excitability.

11. Resting membrane potentials. Membrane potentials of >+30 or >+35 mV are surprisingly positive! Probably these are typos; this could be made clearer with wording such as “more depolarized than.” (“Membrane potentials that are less than” is always a bit ambiguous.)

12. ‘trained RNA/control RNA/vehicle’ would read better with ‘or’ in place of ‘/.’

Results.

13. Training produced “clear long-term sensitization” Did any groups of animals not show healthy sensitization? Were those animals also used for RNA? It would be interesting if poor learners didn't transfer sensitization to naive animals - if this were tested.

14. The Fig. 1 description in Results does not mention an effect of RNA injection on duration of withdrawal compared to the pretest.

15. What amount of RNA was added to culture dishes in what volume? (1 μg in 0.5-1 μL doesn't communicate concentration)

16. S channels and anti-accommodation. This a complex literature. As Goldsmith and Abrams (1992) concluded, there are both steady state and slowly activating S currents, and the modulation of the former accounts for anti-accommodation.

17. In contrast to the ‘classic’ S current described by Klein et al (1982), the change in input resistance is mediated by modulation of steady state S channels. This closure of S channels is not the primary contributor to the modulation by sensitizing stimuli of sensory neuron excitability. The decrease in resting conductance of sensory neurons with 5-HT exposure was detected early, by 1980, but is arguably the least sensitive indicator of long-term plasticity. Given the very modest effects of trained RNA, and the lack of significance in one comparison (Fig. 3D), I would recommend omitting the results on input resistance from this manuscript. If the results are retained, “another parameter of neuronal excitability, input resistance” deserves a reference.

18. The inclusion of results on motor neuron excitability makes little sense. There is not a history of observing increases in motor neuron excitability with long-term sensitization, so why test for this here? If these data are intended as a control, demonstrating the specificity of the effects on sensory neurons of the RNA from trained animals, panels A and B from Fig. 4 should be included in the same figure as panels A and B from Fig. 3.

Discussion.

19. When discussing the arguments that the effect of RNA from trained animals is specific, the lack of effect of the control RNA on the withdrawal response should be emphasized.

20. “Given, as our data indicate, essential components of the engram for LTM in Aplysia can be transferred between conspecifics via RNA, what is the cellular pathway for this transfer?” This sentence, as written, makes little sense. Trizol-extracted RNA injected into the hemocoel may not interact with a normal cellular pathway. Moreover, the rest of the discussion in this paragraph and the discussion of Arc-encapsulated RNA really don't follow from this introductory sentence.

21. “If ncRNAs in the trained RNA entered Aplysia neurons, they likely had multiple

epigenetic effects that contributed to the induction and maintenance of LTM” Why predict multiple epigenetic effects, rather than a single effect?

22. Fig 2 legend. The title “Apparent memory transfer via RNA injection requires DNA methylation” is somewhat confusing. ‘Memory transfer’ is a bit abstract for a figure legend, and ‘apparent’ makes this more confusing. The wording from Results “DNA methylation is required for RNA-induced enhancement of the SWR” would be much clearer.

## Author Response

RESPONSES TO REVIEWERS

Reviewer #1

1. We did not attempt to select a specific subpopulation of sensory neurons for our electrophysiological experiments. But the reviewer raises an interesting point. As we note, the trained RNA produced significant enhancement in a subset of sensorimotor cultures (Fig. 4). In the Discussion we state that a possible reason for this finding is that there is a subset of pleural sensory neurons (or LFS motor neurons) that are particularly prone to facilitation-related modification by the trained RNA.

2. The injected RNA was from multiple donors, typically from 4 sensitized/control animals. The RNA extraction and preparation are now explained in more detail in the Materials and Methods.

3. Done.

4. The Discussion has been shortened significantly (see the response to Reviewer #2's Comment 4 below).

Reviewer #2

Major comments:

1. We have removed the term ‘conspecific’ entirely from the manuscript. We have also eliminated statements about ‘interindividual transfer’ of memory. In addition, in the revised Abstract we have downplayed the finding of memory transfer and emphasized the implication for our findings on the idea that long-term memory is stored at the synapse. We do prefer to retain the reference to memory transfer in the paper's title, however; note that the term ‘memory transfer’ has a significant place in the history of memory research, and we wish our title to refer to the early, somewhat neglected, literature on this subject.

2. We thank the reviewer for his insightful comments on this interesting aspect of our data. We have included an explicit statement that the behavioral changes induced by the trained RNA may be due to changes at loci other than the monosynaptic sensorimotor connections, and, in particular, within interneuronal pathways, and that this may account for the significant difference in the magnitude of the behavioral and cellular changes induced by the trained RNA.

3. Again, we thank the reviewer for his insightful comments on this subject. We have revised our discussion of the synaptic results. We now emphasize that the facilitatory effect of the trained RNA on a subset of synapses may be meaningful. Notice that the relatively modest effect of the trained RNA on sensory neuron excitability might be expected. It is well recognized that dissociated sensory neurons become more excitable due to axonal injury during their removal from the CNS (Bedi and Glanzman, 2001; Weragoda et al., 2004). Thus, the hyperexcitability in the dissociated sensory neurons induced by the trained RNA was no doubt riding on the injury-induced hyperexcitability. The relatively modest effect on sensory neuron excitability induced by the trained RNA may therefore be partly attributable to a ceiling effect. Notice that have been very few assessments of sensory neuron accommodation following the induction of long-term sensitization in intact Aplysia. In the Cleary et al. (1987) study, body wall shock-induced sensitization produced a doubling of the number of sensory neuron action potentials to a 1 s-long current pulse. We observed that the trained RNA produced a 100% increase in the number of evoked spikes compared to the untrained RNA, and a 50% increase compared to the vehicle treatment. When one considers the probable contribution of injury to our accommodation results, unlike in the case of the Cleary et al. study, which measured accommodation in pleural sensory neurons shortly after the pleural ganglia had been removed from intact Aplysia in magnesium chloride, our results are arguably more impressive. In support of this idea, note that Liu et al. (2011) did not observe a significant increase in the excitability of cultured pleural sensory neurons 48 h after long-term training with 5-HT (see Fig. 6 of that study).

4. We have significantly revised the Discussion. In particular, we have shortened the discussion of the historical results regarding memory transfer literature; these results are now summarized in 3 sentences (on pp. 14-15), as the reviewer requested. We have retained the discussion of how naked RNA may have been transferred to neurons in our study but have separated it from the discussion of the early memory transfer results, as the reviewer recommended.

Minor points:

5. The word ‘additional’ has been stricken from this sentence.

6. We have eliminated the information about the specific periods when the animals used in the study were obtained.

7. The intertrain interval was 2 seconds, which is now stated on p. 3.

8. The information regarding the volumes of Trizol used is now provided in the Materials and Methods (pp. 4-5). Note that we have expanded the description of the RNA extraction and preparation to make it easier for the reader to understand this process.

9. The volume of water (DIH2O) used to dissolve the RNA is now stated and the other requested changes made.

10. The recommended changes to the description of the electrophysiological measurements have been made.

11. We have revised the statements regarding the criterion resting membrane potentials for excluding neurons from the electrophysiological tests.

12. The requested change has been made.

13. Yes, as we state, some batches of animals did not exhibit sensitization. No RNA was taken from these poor learners and they were not examined further.

14. We thank the reviewer for pointing this out to us. This finding is now explicitly stated in the Results.

15. The amount of RNA added to the cell cultures is now stated in the Methods and Materials (on p. 7 of the revised manuscript).

16. We now state that the specific potassium channel involved in anti-accomodation is the slowly activating S current, and now cite the Goldsmith and Abrams paper. (Note that the specification by the reviewer that modulation of the ‘former’ S current, i.e., the steady state S current, is responsible for anti-accommodation appears to be a mistake.)

17. We have removed the input resistance data from the paper.

18. The motor neuron excitability data were included to show the cellular specificity of the trained RNA's effect. The sensory and motor neuron excitability have been combined into a single figure, the revised Figure 3, as recommended by the reviewer.

19. Done. See p. 12 of the revised manuscript.

20. This sentence has been removed.

21. We now stated that the trained RNA likely “had one or more epigenetic effects that contributed to the induction and maintenance of LTM (Fig. 2).”

22. The title for Figure 2 has been revised as recommended.
